# Protein S100 as outcome predictor after out-of-hospital cardiac arrest and targeted temperature management at 33 °C and 36 °C

**DOI:** 10.1186/s13054-017-1729-7

**Published:** 2017-06-20

**Authors:** Pascal Stammet, Josef Dankiewicz, Niklas Nielsen, François Fays, Olivier Collignon, Christian Hassager, Michael Wanscher, Johan Undèn, Jorn Wetterslev, Tommaso Pellis, Anders Aneman, Jan Hovdenes, Matt P. Wise, Georges Gilson, David Erlinge, Janneke Horn, Tobias Cronberg, Michael Kuiper, Jesper Kjaergaard, Yvan Gasche, Yvan Devaux, Hans Friberg, Target Temperature Management after Out-of-Hospital Cardiac Arrest (TTM) trial investigators

**Affiliations:** 1 Department of Anesthesia and Intensive Care Medicine, Centre Hospitalier de Luxembourg, 4, rue Barblé, L-1210 Luxembourg, Luxembourg; 2 Department of Cardiology, Skåne University Hospital, Lund, Sweden; 3 Department of Anesthesia and Intensive Care, Helsingborg Hospital, Helsingborg, Sweden; 4 Competence Centre for Methodology and Statistics, Luxembourg Institute of Health, Strassen, Luxembourg; 5 Department of Cardiology B, The Heart Centre, Rigshospitalet University Hospital, Copenhagen, Denmark; 6 Department of Thoracic Anesthesiology, The Heart Centre, Rigshospitalet University Hospital, Copenhagen, Denmark; 7 Department of Anesthesia and Intensive Care, Hallands Hospital, Lund University, Halmstad, Sweden; 8 Copenhagen Trial Unit, Centre of Clinical Intervention Research, Rigshospitalet, Copenhagen, Denmark; 9 Department of Anesthesia and Intensive Care, Azienda Ospedaliera ‘Card. G. Panico’, Tricase, Italy; 10 Department of Intensive Care, Liverpool Hospital, Sydney, NSW Australia; 11 Department of Anesthesia and Intensive Care, Oslo University Hospital, Rikshospitalet, Oslo, Norway; 12 Department of Intensive Care, University Hospital of Wales, Cardiff, UK; 13 Department of Clinical Biology, Centre Hospitalier de Luxembourg, Luxembourg, Luxembourg; 14 Department of Intensive Care, Academic Medical Centrum, Amsterdam, The Netherlands; 15 Section of Neurology, Department of Clinical Sciences Lund, Lund University, Skåne University Hospital, Lund, Sweden; 16 Department of Intensive Care, Leeuwarden Medical Centrum, Leeuwarden, The Netherlands; 17 Department of Intensive Care, Geneva University Hospital, Geneva, Switzerland; 18 Cardiovascular Research Unit, Luxembourg Institute of Health, Luxembourg, Luxembourg; 19 Department of Anesthesia and Intensive Care, Skåne University Hospital, Lund University, Lund, Sweden

**Keywords:** Biomarker, S100, Prognosis, Neuroprognostication, Cerebral performance

## Abstract

**Background:**

We aimed to investigate the diagnostic performance of S100 as an outcome predictor after out-of-hospital cardiac arrest (OHCA) and the potential influence of two target temperatures (33 °C and 36 °C) on serum levels of S100.

**Methods:**

This is a substudy of the Target Temperature Management after Out-of-Hospital Cardiac Arrest (TTM) trial. Serum levels of S100 were measured *a posteriori* in a core laboratory in samples collected at 24, 48, and 72 h after OHCA. Outcome at 6 months was assessed using the Cerebral Performance Categories Scale (CPC 1–2 = good outcome, CPC 3–5 = poor outcome).

**Results:**

We included 687 patients from 29 sites in Europe. Median S100 values were higher in patients with a poor outcome at 24, 48, and 72 h: 0.19 (IQR 0.10–0.49) versus 0.08 (IQR 0.06–0.11) μg/ml, 0.16 (IQR 0.10–0.44) versus 0.07 (IQR 0.06–0.11) μg/L, and 0.13 (IQR 0.08–0.26) versus 0.06 (IQR 0.05–0.09) μg/L (*p* < 0.001), respectively. The ability to predict outcome was best at 24 h with an AUC of 0.80 (95% CI 0.77–0.83). S100 values were higher at 24 and 72 h in the 33 °C group than in the 36 °C group (0.12 [0.07–0.22] versus 0.10 [0.07–0.21] μg/L and 0.09 [0.06–0.17] versus 0.08 [0.05–0.10], respectively) (*p* < 0.02). In multivariable analyses including baseline variables and the allocated target temperature, the addition of S100 improved the AUC from 0.80 to 0.84 (95% CI 0.81–0.87) (*p* < 0.001), but S100 was not an independent outcome predictor. Adding S100 to the same model including neuron-specific enolase (NSE) did not further improve the AUC.

**Conclusions:**

The allocated target temperature did not affect S100 to a clinically relevant degree. High S100 values are predictive of poor outcome but do not add value to present prognostication models with or without NSE. S100 measured at 24 h and afterward is of limited value in clinical outcome prediction after OHCA.

**Trial registration:**

ClinicalTrials.gov identifier: NCT01020916. Registered on 25 November 2009.

**Electronic supplementary material:**

The online version of this article (doi:10.1186/s13054-017-1729-7) contains supplementary material, which is available to authorized users.

## Background

Mortality in comatose out-of-hospital cardiac arrest (OHCA) patients admitted to an intensive care unit (ICU) is around 50%. Whereas initial ICU mortality is caused by hemodynamic failure in the majority of cases, later morbidity and mortality are due mainly to hypoxic brain damage [1, 2]. Withdrawal of life-sustaining therapies (WLST) based on presumed poor neurological prognosis is the predominant cause of death [2, 3]. To better guide therapy and to support decisions on WLST, there is a need for early and accurate outcome prediction tools in this severely ill population.

The S100 protein, a 21 kDa intracellular calcium-binding dimer, is implicated in neuronal differentiation, proliferation, and apoptosis [4]. Many subtypes of the S100 protein are known, but the most studied in humans are the brain-specific homodimers A1B (αβ) and BB (ββ) [5, 6]. S100 is a biomarker candidate for outcome prediction after cardiac arrest (CA) [7, 8], but previous small studies yielded a wide range of cutoff values for a poor outcome, and current guidelines do not advocate its use [9]. S100 is present mainly in white matter, predominantly in astroglial cells, in contrast to neuron-specific enolase (NSE), which is found principally in neurons and neuroendocrine cells [10]. S100 is also commonly present in extracerebral tissues [11, 12]. The Target Temperature Management after Out-of-Hospital Cardiac Arrest (TTM) trial, a multicenter clinical trial that randomized 939 patients to targeted temperature management of 33 °C or 36 °C, provides an opportunity to investigate the role of S100 as a prognostic marker after OHCA [13].

### Goals of this study

The aim of this study was to investigate the diagnostic accuracy of S100 as an outcome predictor after CA and whether serial S100 samples conferred an added value to recommended prognostication models [9]. Another aim was to investigate the potential influence of two target temperatures (33 °C and 36 °C) on S100 release curves.

## Methods

### Study design and setting

All patients included in this study were part of the TTM trial (from November 2010 to July 2013; ClinicalTrials.gov identifier NCT01020916), in which two target temperature regimens were compared in adult unconscious patients admitted to an ICU after an OHCA of a presumed cardiac cause [13]. The TTM trial design, statistical analysis plan, and main results were published previously [13–15]. Patients were randomized to a target temperature of 33 °C or 36 °C. Twenty-eight hours after the start of the intervention, rewarming to 37 °C was started at a maximum speed of 0.5 °C/h. The steering committee approved this predefined substudy before trial completion and before starting analysis of S100.

### Study population

All patients included at sites participating in the biobank substudy of the TTM trial were included. Seven TTM trial sites did not participate in the biobank substudy, owing to logistical issues and legal concerns. Data of patients who died before the scheduled blood sampling and of patients with incomplete sampling were treated as missing.

### Sampling and measurements

After return of spontaneous circulation (ROSC), serum blood samples were collected at 24, 48, and 72 h. All samples were preanalytically processed at the different sites, aliquoted, and frozen at −80 °C before shipment to the Integrated Biobank of Luxembourg. S100 determination was performed 6 months after trial completion at the clinical biology laboratory of the Centre Hospitalier de Luxembourg, and the measurements were therefore not available to the treating physician during the trial.

Determination of S100 (S100A1B and S100BB) was performed using a cobas e601 line with an electrochemiluminescence immunoassay kit (Roche Diagnostics, Rotkreuz, Switzerland). The measurement range extended from 0.005 to 39 μg/L. Samples with values above the measurement range had to be diluted accordingly. Functional sensitivity was set at 0.02 μg/L, and expected normal values were <0.105 μg/L. In our laboratory, between-run precision at concentrations of 0.18 and 2.33 μg/L was 2.6% and 3.6%, respectively.

### Outcomes

We aimed to investigate S100 as a predictor of death and cerebral performance after OHCA in both temperature groups. We defined high S100 cutoff values as having a false-positive rate (FPR) for a poor outcome of ≤5%.

The primary outcome in this study was neurological function at 6 months, dichotomized into good or poor outcome according to the Cerebral Performance Categories Scale (CPC) [16]. The CPC score classifies patients into five categories: CPC 1 (no neurological disability), CPC 2 (minor neurological deficit), CPC 3 (severe neurological impairment, dependent in everyday life), CPC 4 (coma), and CPC 5 (death). CPC scores of 1 or 2 were considered a good outcome, whereas CPC scores of 3–5 were considered a poor outcome. Neurological prognostication as well as WLST were standardized and reported according to the trial protocol [13–15].

### Statistical analysis

All group comparisons of continuous measures were performed using Wilcoxon’s test, whereas the chi-square or Fisher’s exact test was used to assess categorical data. Concentrations of S100 were compared over time using the Wilcoxon signed-rank test.

Univariate analysis consisted of plotting ROC curves of S100 and computing the AUC for each time point. Because there is no established cutoff value for S100 to predict outcome, we took a broad approach in evaluating potential cutoff values. Predictive cutoffs were determined by maximizing the Youden index and by reporting 95–100% specificity for a poor neurological outcome. Multivariable analyses were performed by adding S100 measurements first to a logistic clinical model of CPC adjusted for targeted temperature and for the patients’ characteristics (target temperature, age, time to ROSC, lactate level on admission, sex, bystander CPR, first monitored rhythm, ROSC after bystander CPR and circulatory shock on admission), and then to the same model including both those variables and NSE measurements at 24, 48, and 72 h. Bootstrap internal validation and multiple imputations were further performed to correct sensitivity and specificity, respectively, for optimism and to account for missing data. The continuous Net Reclassification Index (NRI) and the integrated discrimination improvement (IDI) were computed to evaluate the added predictive value of S100. DeLong’s test was used to compare AUCs computed without multiple imputations, and a likelihood ratio test was performed to compare the fit of the models. Differences in survival until the end of the trial were assessed using Kaplan-Meier curves and the log-rank test.

R software (version 2.15.2, http://www.r-project.org/; R Foundation for Statistical Computing, Vienna, Austria) with the packages ROCR, pROC, Hmisc, and rms was used to perform the computations. A *p* value <0.05 was considered statistically significant.

## Results

### Characteristics of study subjects

**Fig. 1:**
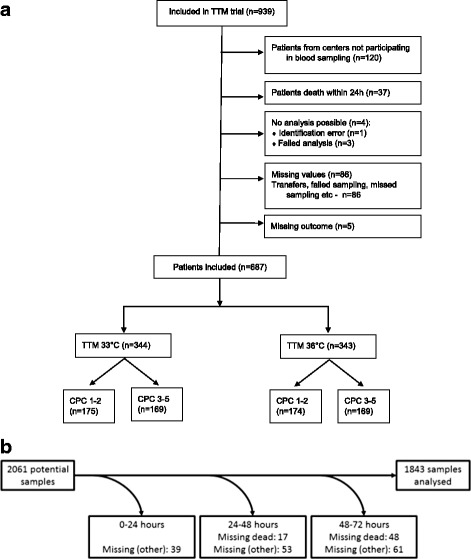
Study flowchart. Number of patients enrolled in the TTM trial and included in this substudy **a**; Number of samples included in this study and reasons for eliminating serum samples from analysis **b**. *TTM* Target Temperature Management after Out-of-Hospital Cardiac Arrest trial, *CPC* Cerebral Performance Categories Scale

The TTM trial researchers investigated 939 patients, who had no difference in mortality or neurological function between the 33 °C and the 36 °C groups [13]. Overall, 700 consecutive patients from 29 different sites participated in the biomarker substudy (Fig. 1a). A total of 1843 serum samples from 687 different patients were analyzed (Fig. 1b). The main patient characteristics are shown in Table 1. There were no marked differences between our study population and the main TTM trial population or in neurological outcome between temperature groups (data not shown).

**Table 1:** Main demographic and Utstein data

	33 °C (*n* = 344)	36 °C (*n* = 343)
Male sex, *n* (%)	292 (83)	273 (79)
Age, mean (SD)	64.2 (11.8)	63.4 (12.9)
First monitored rhythm, *n* (%):		
Asystole or PEA	67 (19)	64 (18)
Non perfusing VT or VF	273 (77)	272 (78)
ROSC after bystander defibrillation	6 (2)	3 (1)
Unknown initial rhythm	6 (2)	8 (2)
Time from CA to ROSC, mean (SD)	30.5 (21.5)	31.1 (23.8)
Lactate, mmol/L, mean (SD)	6.6 (4.4)	6.6 (4.4)
Shock on admission, *n* (%)	45 (13)	43 (12)

*Abbreviations: CA* Cardiac arrest, *CPR* Cardiopulmonary resuscitation, *PEA* Pulseless electrical activity, *ROSC* Return of spontaneous circulation, *VF* Ventricular fibrillation, *VT* Ventricular tachycardia

Values are mean and SD or *n* (%)

### S100 values by outcome group

**Fig. 2:**
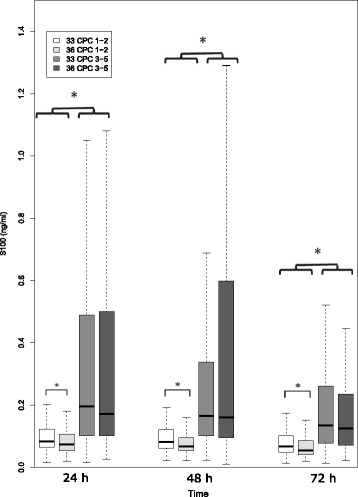
S100 time course. Box plots of S100 over the first 72 h after return of spontaneous circulation. Data are presented as median, quartile 1, quartile 3, and lower fence (i.e., lowest value above [quartile 1–1.5 {quartile3 − quartile1}]) and upper fence (i.e., greater value below [quartile 3 + 1.5 {quartile3 − quartile1}]). A statistical difference was found only for S100 values of patients with good outcomes, with higher values in the 33 °C group and between good and poor outcome groups. * *p* < 0.05. *CPC* Cerebral Performance Categories Scale

Median S100 values were significantly higher in patients with poor versus good outcomes at 24, 48, and 72 h respectively: 0.19 (IQR 0.10–0.49) versus 0.08 (IQR 0.06–0.11) μg/ml, 0.16 (IQR 0.10–0.44) versus 0.07 (IQR 0.06–0.11) μg/L, and 0.13 (IQR 0.08–0.26) versus 0.06 (IQR 0.05–0.09) μg/L (all *p* < 0.001). There was a significant decrease in serum levels in both outcome groups over time (Fig. 2).

### Influence of temperature on S100

S100 values were significantly higher at 24 and 72 h in the 33 °C group than in the 36 °C group (0.12 [0.07–0.22] versus 0.10 [0.07–0.21] μg/L and 0.09 [0.06–0.17] versus 0.08 [0.05–0.10] at 24 and 72 h, respectively; *p* < 0.02). No significant difference was found at 48 h. When comparing the groups by their outcome, we found significantly higher median values in the good outcome groups in the 33 °C arm than in the 36 °C arm: 0.08 (0.07–0.12) versus 0.07 (0.05–0.10) μg/L (*p* = 0.004), 0.08 (0.06–0.12) versus 0.07 (0.05–0.10) μg/L (*p* = 0.002), and 0.07 (0.05–0.10) versus 0.06 (0.04–0.08) μg/L (*p* = 0.002) at 24, 48, and 72 h, respectively. There was no significant difference in levels of S100 between temperature groups in the poor outcome groups.

### Predictive capacity of S100

**Fig. 3:**
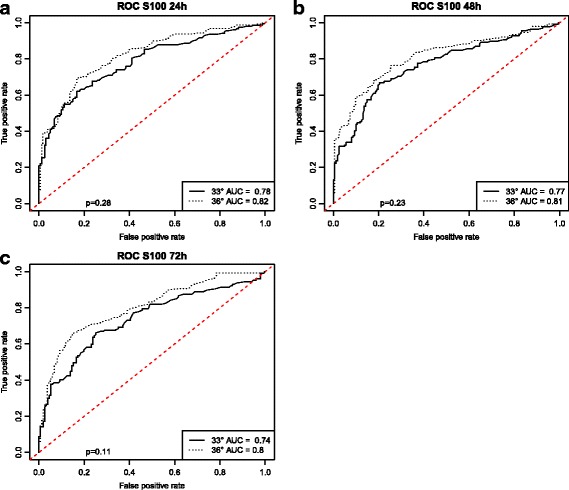
ROC curves with AUCs for S100 at 24 h (**a**), 48 h (**b**), and 72 h (**c**) after return of spontaneous circulation for outcome prediction according to Cerebral Performance Categories Scale score at 6 months

The capacity of S100 to predict CPC score at 6 months was first determined using ROC curves (Fig. 3a–c). The best performance of S100 was at 24 h, with AUCs of 0.78 (95% CI 0.73–0.83) for patients treated at 33 °C and 0.82 (95% CI 0.77–0.87) for patients treated at 36 °C, as well as an AUC of 0.80 (95% CI 0.77–0.83) when both temperature groups were combined. At 48 h and 72 h, AUCs were lower. AUCs did not differ significantly between temperature groups at any time point (*p* > 0.11).

Cutoff values with FPRs ranging from 0 (100% specificity) to 5%, as well as with a maximized Youden index for all patients, are presented in Table 2. Cutoff values for both temperatures groups were not markedly different, except for those with an FPR of 0 (data not shown).

**Table 2:** S100 cutoff values

Time point		Cutoff (μg/L)	Sensitivity	95% CI	Specificity	95% CI
	S100 Youden	0.12	0.68	0.63–0.73	0.77	0.73–0.82
	S100_5	0.25	0.41	0.35–0.46	0.95	0.93–0.97
	S100_4	0.28	0.40	0.34–0.45	0.96	0.94–0.98
24 h	S100_3	0.32	0.35	0.30–0.40	0.97	0.95–0.99
	S100_2	0.36	0.32	0.26–0.37	0.98	0.96–0.99
	S100_1	0.72	0.22	0.17–0.26	0.99	0.97–1.00
	S100_0	2.59	0.10	0.07–0.13	1.00	0.99–1.00
	S100 Youden	0.13	0.63	0.57–0.68	0.82	0.78–0.86
	S100_5	0.25	0.36	0.30–0.41	0.95	0.93–0.98
	S100_4	0.25	0.36	0.30–0.41	0.96	0.94–0.98
48 h	S100_3	0.27	0.34	0.28–0.39	0.97	0.95–0.99
	S100_2	0.28	0.34	0.28–0.39	0.98	0.96–0.99
	S100_1	0.36	0.28	0.23–0.34	0.99	0.97–0.99
	S100_0	3.67	0.05	0.03–0.08	1.00	0.99–1.00
	S100 Youden	0.10	0.65	0.59–0.71	0.80	0.75–0.84
	S100_5	0.19	0.35	0.29–0.40	0.95	0.92–0.97
	S100_4	0.23	0.29	0.24–0.35	0.96	0.94–0.98
72 h	S100_3	0.26	0.25	0.20–0.30	0.97	0.95–0.99
	S100_2	0.35	0.20	0.15–0.24	0.98	0.96–0.99
	S100_1	0.52	0.15	0.11–0.19	0.99	0.97–0.99
	S100_0	1.83	0.05	0.02–0.08	1.00	0.98–1.00

S100 cutoff values for poor outcome prediction, pooled data for target temperature

S100 Youden indicates S100 cutoff with the compromise of the best sensitivity and specificity (maximized Youden index). The number following S100 refers to the false-positive rate. Sensitivity and specificity are corrected by bootstrap internal validation

**Fig. 4:**
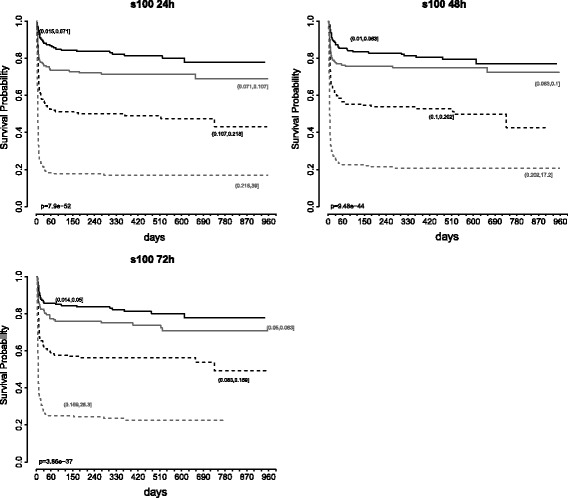
Kaplan-Meier curves for prediction of survival at the end of the trial (primary endpoint of the Target Temperature Management after Out-of-Hospital Cardiac Arrest trial) for S100 values at 24 h (**a**), 48 h (**b**), and 72 h (**c**) after return of spontaneous circulation. Separation into quartiles of serum S100 levels

Survival was associated with S100 levels and was significantly lower in groups with higher S100 levels as defined by quartiles (Fig. 4). At each time point, S100 was a significant predictor of survival in both temperature groups (*p* < 0.001).

### Multivariable analysis

In multivariable analysis including the allocated target temperature and baseline variables (age, sex, bystander cardiopulmonary resuscitation, first monitored rhythm, time to ROSC, lactate levels on admission, and circulatory shock), all variables except target temperature, gender and shock on admission were independent neurological outcome predictors (AUC 0.80, 95% CI: 0.76–0.83, sensitivity 0.73, specificity 0.76) (data not shown). When serial S100 values were added to this model, none of the three S100-measurements was an independent outcome predictor, (Table 3) but the AUC of the model including serial samples improved to 0.84 (95%CI: 0.81–0.87, sensitivity 0.75, specificity 0.81, DeLong test *p* < 0.001, likelihood test *p* < 0.001). Adding S100 improved the reclassification of patients significantly as demonstrated by continuous NRI (0.53, *p* < 0.001) and IDI (0.08, *p* < 0.001). When adding serial S100 values to another, previously published model including the same clinical characteristics and NSE values at the 3 time-points (AUC 0.92, 95%CI: 0.90–0.94) [17], S100 did not further improve the AUC (0.92, 95%CI: 0.90–0.94, sensitivity 0.81, specificity 0.92, DeLong test *p* = 0.13, likelihood test *p* = 0.08) (Table 4).

**Table 3:** Multivariable analysis with multiple imputation: clinical variables and S100

			95% CI		
S100 + clinical	Effect	Odds ratio	Lower	Upper	*p* Value
Intercept	−3.670		0.01	0.11	<0.001
S100 at 24 h	1.828	6.221	0.77	50.55	0.09
S100 at 48 h	0.873	2.395	0.13	45.81	0.56
S100 at 72 h	1.594	4.926	0.21	117.39	0.32
Target temperature	0.085	1.089	0.75	1.59	0.66
Age	0.062	1.064	1.05	1.08	<0.001
Time CA to ROSC	0.022	1.022	1.01	1.03	<0.001
Lactate level on admission	−0.001	0.999	0.95	1.05	0.98
Sex	−0.271	0.762	0.47	1.24	0.27
Bystander CPR performed	−0.527	0.590	0.39	0.90	0.02
VT/VF versus PEA/asystole	−1.431	0.239	0.13	0.43	<0.001
ROSC after bystander defibrillation	−1.560	0.210	0.05	0.88	0.03
Shock on admission	0.160	1.173	0.62	2.21	0.98

*Abbreviations: CA* Cardiac arrest, *CPR* Cardiopulmonary resuscitation, *PEA* Pulseless electrical activity, *ROSC* Return of spontaneous circulation, *VF* Ventricular fibrillation, *VT* Ventricular tachycardia

**Table 4:** Multivariable analysis with multiple imputation of clinical variables, S100, and neuron-specific enolase

			95% CI		
Model S100 + NSE + clinical analysis	Effect	Odds ratio	Lower	Upper	*p* Value
Intercept	−6.480		0.00	0.01	<0.001
S100 at 24 h	1.012	2.751	0.49	15.33	0.25
S100 at 48 h	−1.808	0.164	0.00	6.89	0.34
S100 at 72 h	2.284	9.820	0.24	401.61	0.23
NSE at 24 h	−0.041	0.960	0.93	0.98	<0.001
NSE at 48 h	0.065	1.068	1.04	1.10	<0.001
NSE at 72 h	0.026	1.026	1.00	1.05	0.02
Target temperature	0.187	1.206	0.76	1.91	0.43
Age	0.091	1.095	1.07	1.12	<0.001
Time CA to ROSC	0.009	1.010	1.00	1.02	0.17
Lactate level on admission	0.003	1.003	0.94	1.07	0.93
Sex	−0.400	0.671	0.38	1.20	0.18
Bystander CPR performed	−0.706	0.494	0.29	0.83	0.01
VT/VF versus PEA/asystole	−1.062	0.346	0.17	0.72	<0.001
ROSC after bystander defibrillation	−0.926	0.396	0.07	2.11	0.28
Shock on admission	0.356	1.428	0.68	2.99	0.34

*Abbreviations: CA* Cardiac arrest, *CPR* Cardiopulmonary resuscitation, *NSE* Neuron-specific enolase, *PEA* Pulseless electrical activity, *ROSC* Return of spontaneous circulation, *VF* Ventricular fibrillation, *VT* Ventricular tachycardia

We thereafter repeated the same multivariable analysis without multiple imputations and in unconscious patients on day 3, with and without the addition of NSE. In each analysis, S100 was not an independent outcome predictor.

## Discussion

In this substudy of a large international trial, the use of S100 for outcome prediction after OHCA was assessed. S100 values were higher in patients with poor outcomes at all time points, with the best capacity for S100 to predict outcome being at 24 h. In multivariable analysis, S100 measurements at 24, 48, and 72 h were not significant predictors of outcome. The joint effect of the three measurements, however, improved the AUC, NRI, and IDI of a predictive model that included established clinical characteristics associated with outcome.

In previous, smaller studies, researchers compared S100 in two target temperature groups and could not detect a significant influence of temperature on S100 levels [18, 19]. In this study, S100 values were higher at 24 and 72 h in the 33 °C group than in the 36 °C group, which was explained by higher S100 values among patients with good outcomes in the 33 °C group. Because the intervention groups and their outcomes were very similar in all aspects other than the intervention temperature, we speculate that this difference might be related to the targeted temperature. In addition, the observed values among patients with good outcomes were well below the suggested cutoff levels for S100. Although we do not have a clear explanation for this result, we consider the finding to be of negligible clinical relevance.

S100 could distinguish patients with good and poor outcomes after OHCA because median values were higher in the poor outcome group, and this has been described in previous reports [18, 20–25]. It is noteworthy that S100 values declined over time in both temperature groups and for both outcome groups, indicating an early peak of this biomarker, which might explain why the first sample (at 24 h after ROSC) showed the best results [26]. We did not collect blood samples before 24 h after ROSC, and higher levels prior to 24 h cannot be ruled out. However, a clear peak earlier than 24 h could not be determined in a previous study in which researchers investigated the kinetic profile of S100 [23]. Other studies have also confirmed a similar decline over time after 24 h in patients with good and poor outcomes [22, 25]. The early release and subsequent decline may be explained by the short half-life of approximately 2 h in combination with a low molecular weight, allowing a rapid transition through the blood-brain barrier [27]. This differentiates S100 from other biomarkers (e.g., NSE), where the kinetics between 24 and 72 h after CA are indicative of outcome [17]. The earlier peak of S100 and its relative strength over NSE and other biomarkers for outcome prediction at 24 h could potentially be of clinical use under certain circumstances, such as when prolonged care after rewarming might be considered unethical and several prognostic indicators point to a poor outcome. Another argument in favor of using S100 as an adjunct in prognostication after CA might be its availability in many centers, owing to its common use in the assessment of traumatic brain injury [28].

The cutoff values for S100 in this study are comparable with those described previously [7, 8, 25, 29]. Any differences might be due to different assays that might yield different values [20, 22, 23], different outcome measures [29], and sample size [23]. As with other biomarkers, an absolute cutoff value with an FPR of 0 for poor outcome may be unrealistic and would limit its use. A more feasible approach might be to choose a higher FPR, which might be acceptable when used in combination with other prediction tools [9]. In this study, a cutoff with an FPR of 5% would correspond to an S100 serum level of 0.25 μg/L at 24 h after ROSC.

As with any other prognostication method, prediction should be based on a protocol including a holistic approach and with multiple tests and parameters [9, 30]. Clearly, NSE outperformed S100 for outcome prediction after CA in the same patient cohort [17]. Adding S100 to our model including clinical characteristics and NSE did not further improve the accuracy of the model. Similar results have also been described by others when S100 was added to NSE [25]. Using a multivariable model with fewer variables, researchers in another study suggested the usefulness of S100 over NSE on admission [22]. Although the use of a combination of biomarkers for outcome prediction is intriguing, we failed to demonstrate any added value of S100 in a clinical model including NSE.

### Limitations and strengths

Biomarkers are unlikely to be affected by sedation, in contrast to some neurophysiological tests or the clinical examination, and therefore they may be more objective markers of brain injury. However, they are measured intermittently, whereas their production or secretion and metabolism are a dynamic process, underscoring the importance of serial measurements. This study is a predefined substudy of the TTM trial, and we acknowledge any potential limitations of this trial. Not all patients included in the TTM trial participated in the sampling, and not all patients had a sample drawn at each time point. Because of randomization stratified by site, we believe that this did not have a significant influence on the results and that there was no difference between our study cohort and the main TTM trial cohort. We acknowledge that, according to our study protocol, there was no blood sampling on admission or prior to 24 h, which deprived us from analyzing the potential value of very early S100 measurements. Another limitation is that we had no external quality control at the participating sites where samples were collected and preanalytically processed.

The main strength of our study is the large sample size of a predefined substudy of a multicenter clinical trial investigating two target temperatures in comatose patients after OHCA. The TTM trial had strict rules and protocols regarding prognostication and how WLST was conducted [14]. In addition, all the samples were analyzed at a single core laboratory after the completion of the study, ruling out the problem of variation between laboratories and limiting the risk of “self-fulfilling prophecy” due to having bedside access to the biomarkers.

## Conclusions

There was no clinically important effect of two different target temperatures on levels of S100. High S100 values are predictive of poor outcome after OHCA but do not add any real value to present prognostication models with or without NSE. S100 measured at 24 h and afterward is of limited value in clinical outcome prediction after OHCA, especially in a setting where NSE is available.

## Additional file

Additional file 1: List of the ethical review boards that accepted the trial and the approval reference numbers. (DOCX 12 kb)

## Abbreviations

CA: Cardiac arrest
CPC: Cerebral Performance Categories Scale
CPR: Cardiopulmonary resuscitation
FPR: False-positive rate
ICU: Intensive care unit
IDI: Integrated discrimination improvement
NRI: Net Reclassification Index
NSE: Neuron-specific enolase
OHCA: Out-of-hospital cardiac arrest
PEA: Pulseless electrical activity
ROSC: Return of spontaneous circulation
TTM: Target Temperature Management after Out-of-Hospital Cardiac Arrest trial
VF: Ventricular fibrillation
VT: Ventricular tachycardia
WLST: Withdrawal of life-supporting therapies
