# Supplementary material for: Protein S100 as outcome predictor after out-of-hospital cardiac arrest and targeted temperature management at 33 °C and 36 °C
Source: Crit Care. 2017 Jun 20;21:153. doi: 10.1186/s13054-017-1729-7 (PMC5477102; doi:10.1186/s13054-017-1729-7)
Supplement: Additional file 1: — List of the ethical review boards that accepted the trial and the approval reference numbers. (DOCX 12 kb) [file 13054_2017_1729_MOESM1_ESM.docx]

**Ethical approvals**

List of the ethic review boards that accepted the trial and the ID-references of approval

**Australia**: Health Ethics Review Committee Protocol No X11-0150 & HREC/11/RPAH/216 –

“GI-CCT886

**Czech Republic**: Ethics committee of the General University Hospital of Prague, c/j 193-11 S 17.2.2011

**Denmark**: De vitenskabsetiske Komiteer i Region Hovedstaden, H-1-2010-059

**Italy**: Comitato Etico Indipendente, Hospedaliera S Maria degli Angeli Pordenone, No 9

**Luxembourg**: Comité National d’Ethique de Recherche CNER No 201007/05 Ver 1.0

**The Netherlands**: Medisch Etische Toetsingscommissie MEC 10/107 # 10.17.0921

**Norway**: Regional komité for medisinsk och helsefaglig forskningsetikk Sør-øst C Ref

2010/384

**Sweden**: Regional Ethical Review Board Lund, Protocol 2009/6 Dnr 2009/324 (TTM-Trial)

**Switzerland**: Comité d’Ethique de Recherche CER 10-254 (NAC 10-088)

**United Kingdom**: Cardiff and Vale Research Review Service, Project ID 10/AIC/4927,

Research Ethics Committee for Wales: 10/MRE09/41
